# Real-time trajectory imaging of alpha particles emitted from actinium-225 and its daughter radionuclides

**DOI:** 10.1038/s41598-025-87014-7

**Published:** 2025-01-20

**Authors:** Seiichi Yamamoto, Masao Yoshino, Kenji Shirasaki, Kohei Nakanishi, Kei Kamada, Akira Yoshikawa, Jun Kataoka

**Affiliations:** 1 Faculty of Science and Engineering, Waseda University, Tokyo, Japan; 2 New Industry Creation Hatchery Center, Tohoku University, Sendai, Japan; 3 Institute of Material Research, Tohoku University, Sendai, Japan; 4 Nagoya University Graduate School of Medicine, Nagoya, Japan

**Keywords:** Alpha particles, Actinium-225, Trajectory, Imaging, energy spectrum, Applied physics, Biological physics, Nuclear physics, Optical physics, Particle physics, Techniques and instrumentation

## Abstract

In targeted alpha-particle therapy, actinium-225 (Ac-225) has emerged as a radionuclide of potential, driving extensive efforts to develop innovative radiopharmaceuticals. High-resolution imaging of alpha particles is required for precisely detecting alpha-emitting radionuclides in cellular environments and small organs. Here, we report real-time trajectory imaging of alpha particles emitted by Ac-225 and its daughter radionuclides, utilizing an alpha particle trajectory imaging system. This system incorporates a magnification unit, a cooled electron-multiplying charge-coupled device (EM-CCD) camera, and a Ce-doped Gd_3_Al_2_Ga_3_O_12_ (GAGG) scintillator. Alpha particles were projected onto the GAGG scintillator, producing magnified images that were captured at 100 ms intervals. We successfully tracked particle trajectories with varying lengths and intensities for 4 different alpha particles emitted from Ac-225 and its daughter radionuclides with a spatial resolution of 1.0 μm. Notably, we achieved the imaging of sequentially emitted trajectories from Fr-221 and its decay product At-217, characterized by short decay intervals, along with the extended trajectories of high-energy alpha particles emitted by Po-213. These results demonstrate that high-resolution trajectory imaging, integrated with temporal and energy information, offers profound insights into the real-time behavior of Ac-225 and its daughter radionuclides within living cells or tissue sections, thereby driving advancements in targeted alpha-particle therapy.

**Supplementary Information:**

The online version contains supplementary material available at 10.1038/s41598-025-87014-7.

## Introduction

In targeted alpha-particle therapy, actinium-225 (Ac-225) has emerged as a prominent radionuclide due to its potential for clinical application in therapeutic settings. Ac-225 undergoes decay with the emission of four alpha particles and possesses a relatively long half-life of approximately 10 days. This unique decay profile induces double-stranded DNA damage, thereby rendering Ac-225 highly effective in the destruction of tumor cells^1^. Following promising reports regarding the clinical efficacy of Ac-225 in therapies targeting prostate-specific membrane antigen (PSMA)^2^, pharmaceutical companies have initiated clinical trials^3^, and significant efforts and investments are underway to develop novel radiopharmaceuticals that leverage the properties of Ac-225^4^.

High-resolution imaging of alpha particles is crucial for the detection of Ac-225-based compounds within cellular environments or small organs. Such imaging is instrumental for accurately mapping the distribution of these compounds in cells or dissected animal models, thereby facilitating the advancement of new radiopharmaceuticals and enhancing dosimetry for targeted alpha-particle therapy^5,6^. Achieving high spatial resolution in real-time requires the integration of photodetectors with scintillators to construct effective alpha-particle imaging systems^7–11^. However, many existing systems often fall short in delivering real-time imaging of alpha particle trajectories. While plastic materials, such as nuclear emulsion plates or CR-39 film, are capable of achieving excellent spatial resolution and capturing alpha particle trajectories, they do not support real-time measurements. Furthermore, film-based imaging systems necessitate extensive post-processing, including film development and microscopic examination of trajectories, a process that is both time-consuming and labor-intensive^5^.

Recently, we have developed a high-resolution, high-efficiency evaluation system for scintillators utilized in X-ray imaging^12^. This system can attain spatial resolutions of up to 1 μm when coupled with micro-focus X-rays or synchrotron radiation. We have successfully adapted this system for imaging alpha particle trajectories from americium-241 (Am-241)^13^. We posit that this trajectory imaging system will also be applicable for imaging clinically significant alpha-emitting radionuclides, such as Ac-225, which features a suitable half-life and emits four alpha particles with diverse decay times and energies. If the trajectories of alpha particles emitted from Ac-225 and its daughter radionuclides could be imaged, real-time visualization of alpha particle trajectories within cells or tissue sections will become possible. This advancement would enable more precise, reliable, and effective microscopic analysis of alpha-emitting radionuclides used in therapy.

In this paper, we present the real-time imaging results of alpha particles trajectories from Ac-225 and its daughter radionuclides, achieving a spatial resolution of approximately 1 μm and a temporal resolution of up to 100 ms, complemented by energy information. These results will open the door to the new microscopic analysis of alpha-emitting radionuclides used in therapy.

## Methods

1. Ac-225 source used for experiments.

In this study, we employed a solution of actinium-225 (Ac-225) isolated from uranium-233 (U-233) at the Institute for Materials Research, Tohoku University. The Ac-225 dissolved HCl solution was deposited onto a glass plate and subsequently dried. The radioactivity of the Ac-225 utilized for imaging was approximately 400 kBq over an area of approximately 2 cm^2^. This Ac-225 source facilitated experiments utilizing a 20x lens for imaging at 500 ms intervals.

For imaging experiments conducted at 100 ms intervals with a 40x lens, to reduce the distance between alpha source and scintillator plate minimum, Ac-225 with a radioactivity of approximately 30 Bq was adhered to the scintillator plate, which comprised the field of view of the imaging system, and was then dried to decrease the attenuation of the alpha particle’s energy.

**Fig. 1:**
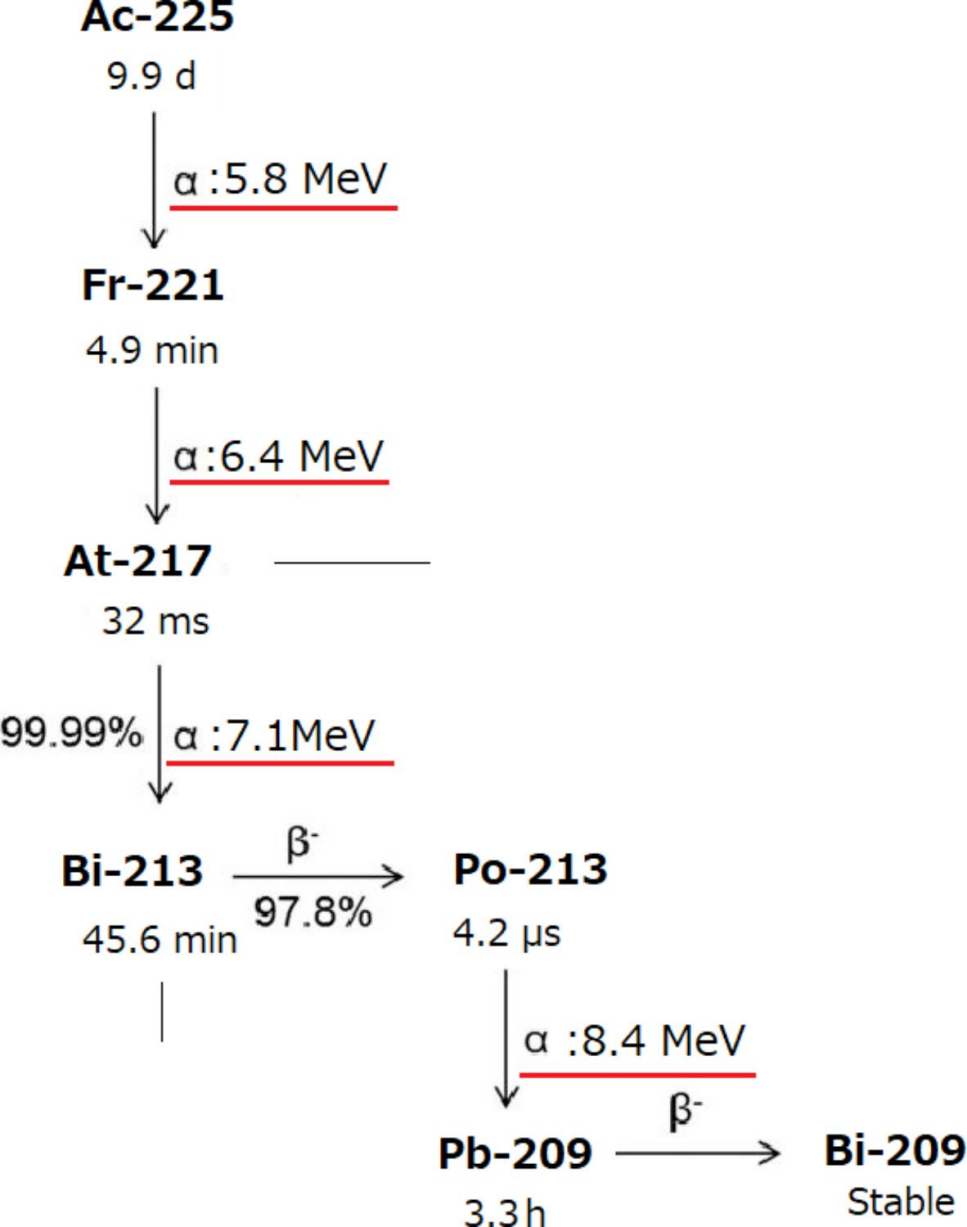
Half-life and alpha particle energies of Ac-225 and daughter radionuclides.

Figure 1 presents the decay scheme of Ac-225 and its daughter radionuclides^14,15^, detailing the energies of the emitted alpha particles alongside their respective half-lives. Ac-225 and its daughter radionuclides primarily yield four alpha particles throughout their decay chains. Notably, the 6.4 MeV alpha particles from francium-221 (Fr-221) and the 7.1 MeV alpha particles from astatine-217 (At-217) are emitted in close temporal proximity, within a half-life of 32 ms, suggesting the potential to image these trajectories as originating from a single radionuclide. Additionally, polonium-213 (Po-213) emits alpha particles with the highest energy (8.4 MeV), which could enable the visualization of extended particle trajectories.

(2) Energy spectra measurement of alpha-particles emitted from Ac-225 daughter radionuclides with silicon charged-particle detector.

To confirm that the Ac-255 source achieved secular equilibrium, we measured the energy spectra of alpha particles emitted from the Ac-225 and its decayed products used in the experiments. This was conducted using a silicon charged-particle detector (ORTEC, USA) in conjunction with an alpha spectroscopy system (Seiko EG&G, Japan). The Ac-225 sample, along with its daughter radionuclides, was positioned within the alpha spectroscopy system, which was then evacuated, allowing for the measurement of the energy spectra of the emitted alpha particles.

(3) Trajectory imaging of alpha-particles emitted from Ac-225 and its daughter radionuclides.

**Fig. 2:**
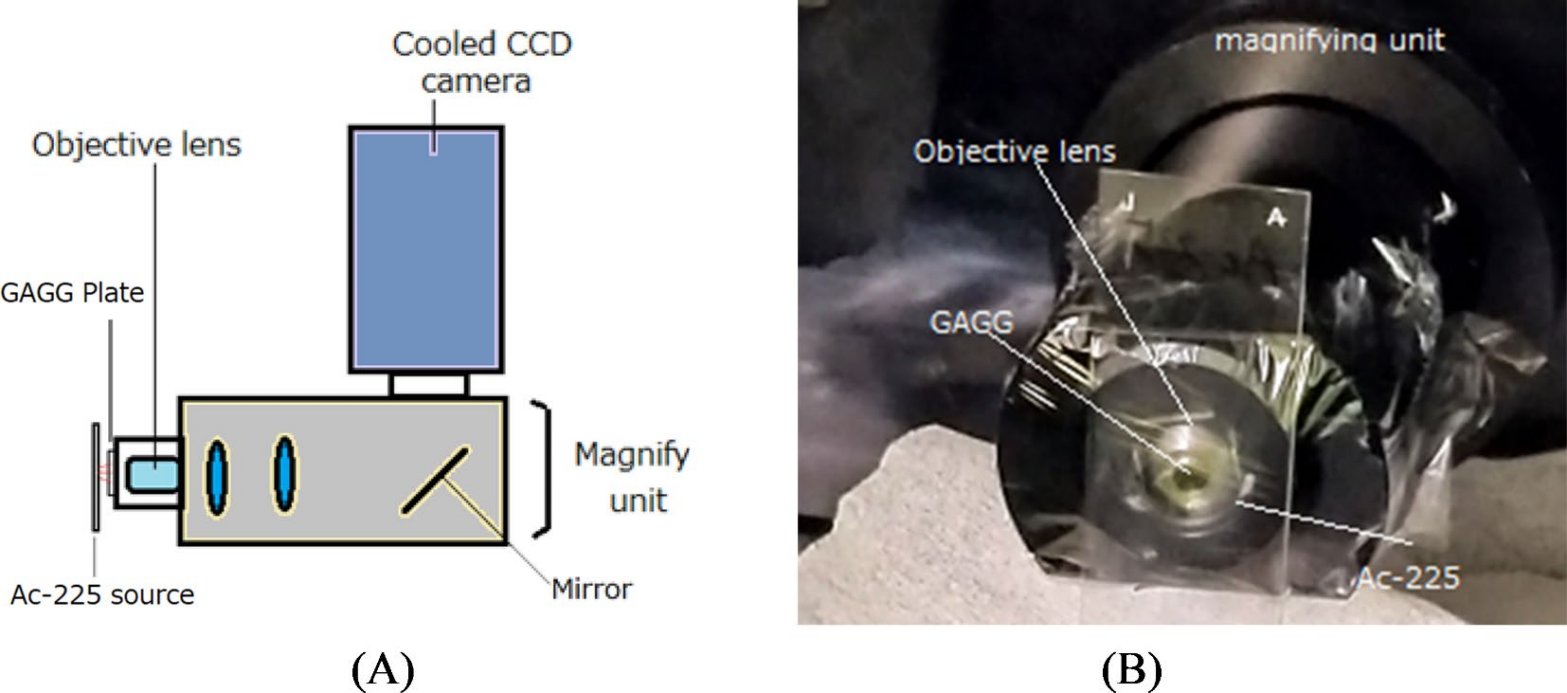
Schematic drawing of developed high-resolution alpha-particle imaging system (**A**) and photo during imaging (**B**).

A schematic illustration of the developed high-resolution alpha-particle imaging system is shown in Fig. 2A. The system consists of a magnifying unit and an electron-multiplying charge-coupled device (EM-CCD) camera, integrated with a 100 μm thick Ce-doped Gd_3_Al_2_Ga_3_O_12_(GAGG) scintillator plate^13^. GAGG was selected as the scintillator material due to its transparency, high light output, and emission spectra that are compatible with CCD cameras. The key characteristics of GAGG include a density of 6.63 g/cm³, a maximum emission wavelength of 520 nm, and a light output ranging from 45,000 to 50,000 photons per MeV.

Alpha particles emitted from Ac-225 and its daughter radionuclides were directed onto the GAGG scintillator plate, as shown in Fig. 2B. The scintillation light produced in the GAGG plate was magnified by the unit, reflected by a mirror within the unit, and subsequently captured by the EM-CCD camera positioned above the magnifying assembly. The magnifying unit employed in this imaging system is a commercial model (AA51, Hamamatsu Photonics, Japan) and is equipped with either a 40× objective lens (CFI Plan Apo Lambda 40×, Nikon Corporation, Tokyo, Japan) or a 20× objective lens (CFI Plan Apo Lambda 20×, Nikon Corporation, Tokyo, Japan).

The GAGG plate was set directly in front of the objective lens. The camera utilized for the alpha-particle imaging system was a cooled electron-multiplying charge-coupled device (EM-CCD) model, operating at −65 °C (Hamamatsu Photonics, ImagEM C9100, Japan). This EM-CCD sensor featured a pixel matrix of 512 × 512. The entire imaging system was enclosed in a light-shielded black box to facilitate seamless operation during experiments.

A desktop computer was employed to control the EM-CCD camera and to display the real-time alpha particle images captured from outside the black box. The focus of the magnifying unit could be adjusted externally with motor control, enabling precise focusing of the alpha particle images on the GAGG plate. The field of view of the EM-CCD with the 40× lens was 200 μm × 200 μm, with a pixel size of 0.4 μm × 0.4 μm. When using the 20× lens, the FOV increased to 400 μm × 400 μm, with a pixel size of 0.8 μm × 0.8 μm.

Trajectory imaging was conducted during the irradiation of alpha particles using the EM-CCD camera with the 40× lens using the GAGG plate directly coated with the Ac-225 solution. Multiple images were captured with the acquisition time of 100 ms and stored on the desktop computer. Additionally, to derive energy spectra, trajectory imaging was performed with the 20× lens to capture a greater number of trajectories in an image to evaluate the energy spectra of alpha particles. The glass plate containing the Ac-225 source was positioned 5 mm from the GAGG plate and measured with an acquisition time of 500 ms, with multiple images acquired and stored on the desktop computer.

(4) Image processing.

The images captured by the EM-CCD camera were processed using the ImageJ software application^16^. The acquired images were stacked, and a smoothed blank image was subtracted to correct for non-uniform background levels. To assess spatial resolution and particle range, profiles were generated from the images containing alpha particle trajectories. These profiles were then analyzed using a Gaussian-fit function in Origin 2018b software^17^, enabling precise determination of their widths.

(5) Monte Carlo simulation.

We simulated the trajectory images and energy spectra of alpha particles emitted from Ac-225 and its daughter radionuclides in the GAGG scintillator using the Monte Carlo simulation method, implemented through the Geant4 toolkit (version 10.7, patch 2)^18^. The simulation was designed to replicate the dimensions and conditions of the experimental setup employed in the trajectory imaging system. The simulated trajectory images were then compared with the experimentally obtained images, and were further used to assess the energy spectra and the ranges of the alpha particle trajectories.

## Results

1. Energy spectra measurement of alpha-particles emitted from Ac-225 and its daughter radionuclides with silicon charged-particle detector.

**Fig. 3:**
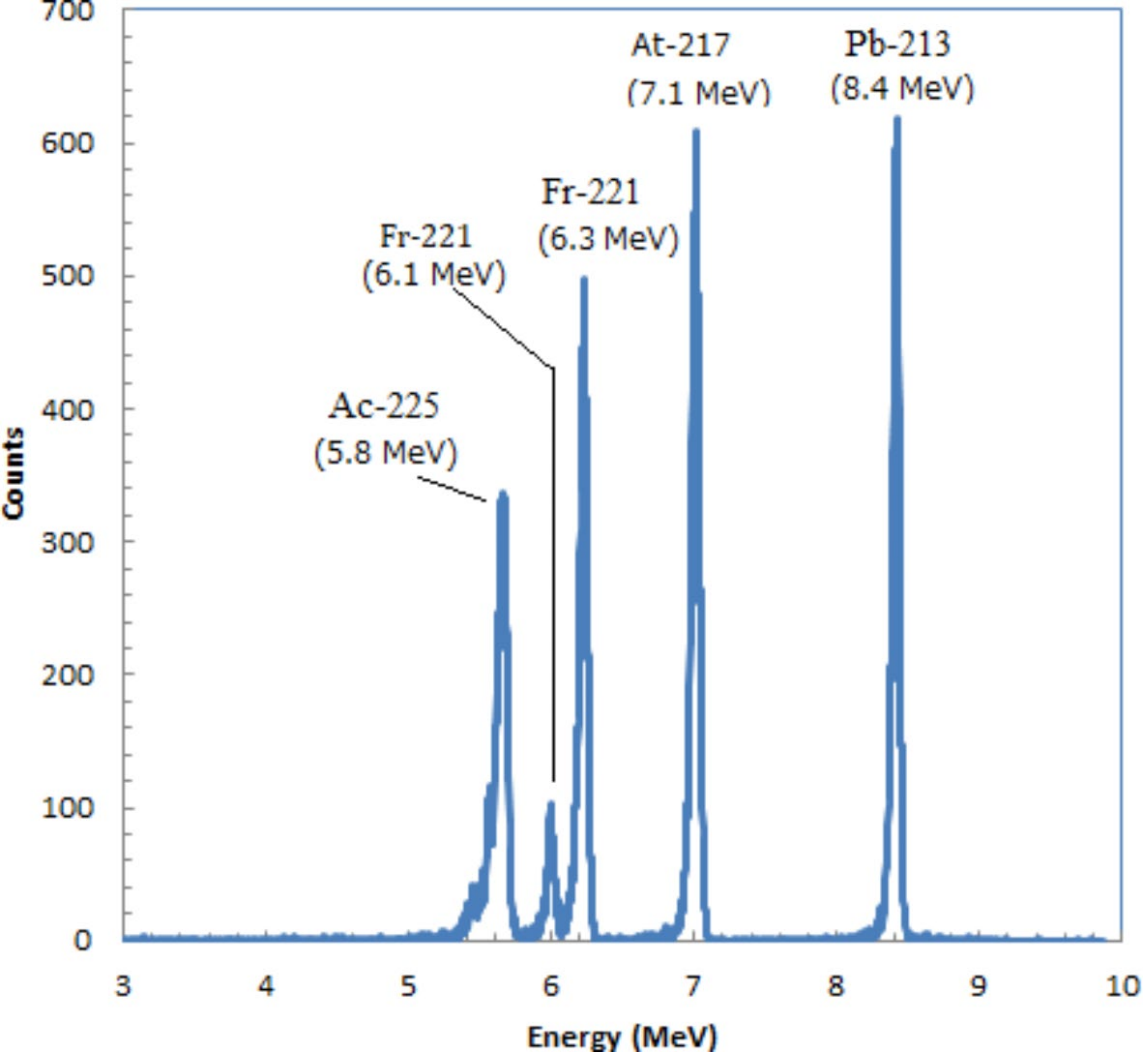
Energy spectra measurement of alpha-particles emitted from Ac-225 and its daughter radionuclides measured with silicon charged-particle detector.

The energy spectra of alpha particles emitted from Ac-225 and its daughter radionuclides, measured using a silicon charged-particle detector, are presented in Fig. 3. Prominent peaks were observed corresponding to Ac-225 (5.8 MeV), Fr-221 (6.3 MeV), At-217 (7.1 MeV), and Po-213 (8.4 MeV). The small peak at 6.1 MeV, between the 5.8 MeV and 6.3 MeV peaks, is another alpha particle emission from Fr-221, with an emission fraction of 15.1%. The spectra indicated four high-count peaks from Ac-225 and its daughter radionuclides, reflecting a radiative equilibrium, which is consistent with previous reports^19,20^.

(2) Trajectory imaging of alpha-particles emitted from Ac-225 and its daughter radionuclides.

**Fig. 4:**
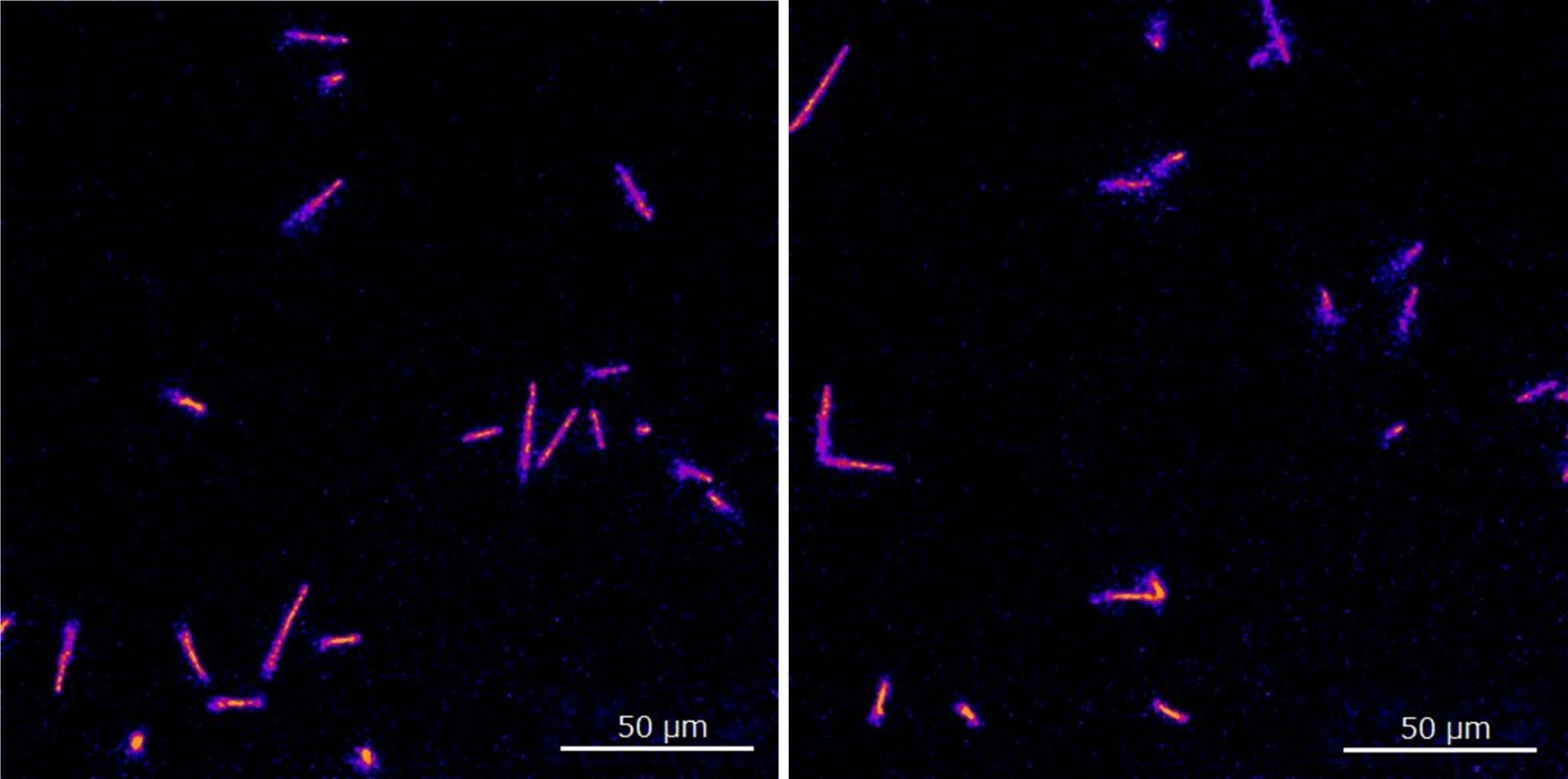
Two composite images each created by summing 10 individual frames captured over 100 ms with a 40× lens.

Two composite images, each created by summing 10 individual frames captured over 100 ms with a 40× lens, are presented in Fig. 4. These images similarly show alpha particle trajectories of different lengths and intensities.

Additionally, a video featuring the 100 ms acquisition time images captured with a 40× lens, illustrating alpha particles emitted from Ac-225 and its daughter radionuclides within the GAGG plate, is provided in Supplemental Material-1. The video was displayed 5 times slower (2 frames per sec) to be observed details.

**Fig. 5:**
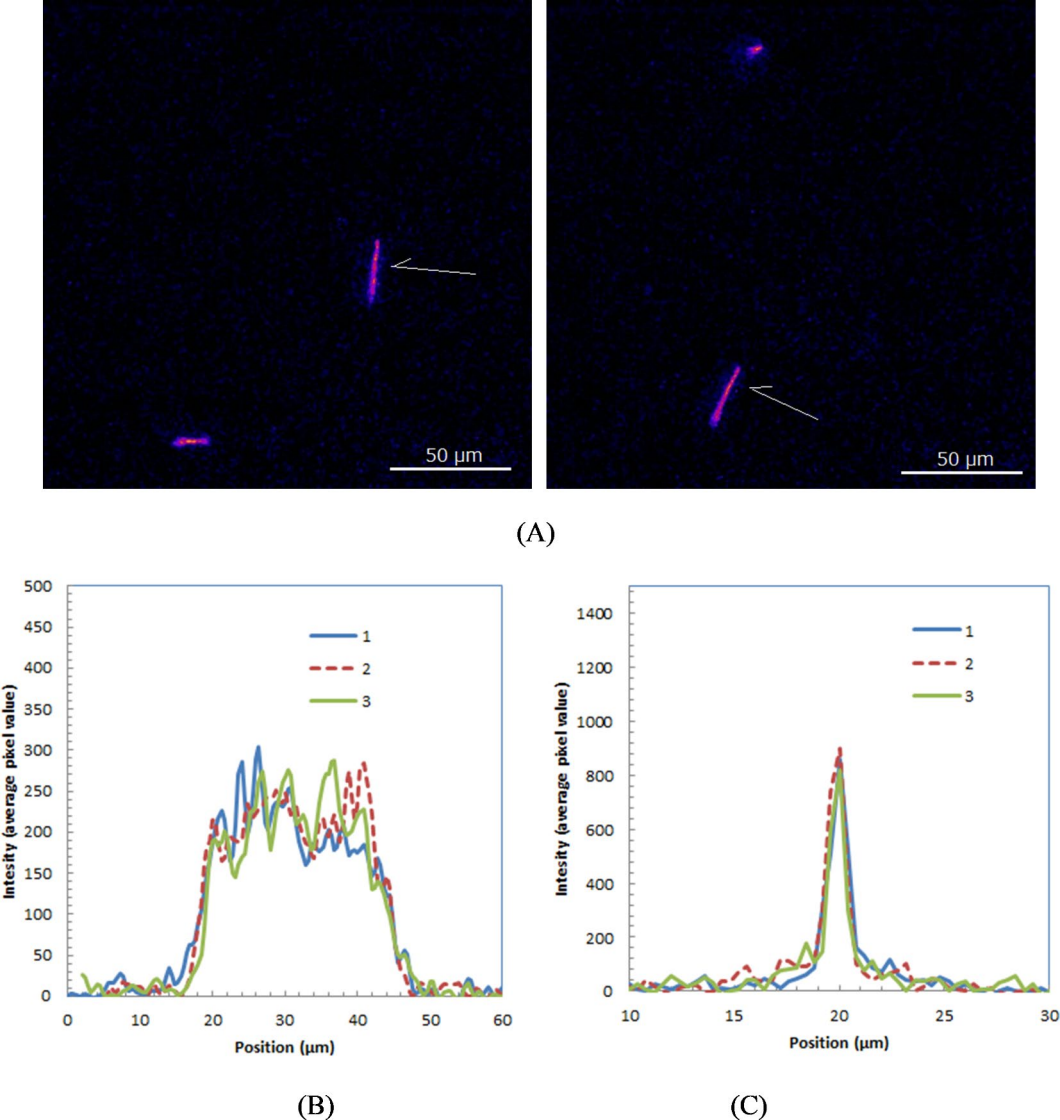
Two of measured images containing alpha particles from Po-214 (8.4 MeV) (indicated with arrow) measured with 100 ms and 40 x lens (**A**) and depth (**B**) and lateral profiles (**C**) of alpha particles.

Two images, each capturing a longest alpha particle trajectory measured with a 40× lens, are shown in Fig. 5A. These alpha particles, which entered the GAGG plate almost parallel to its surface, are identified as 8.4 MeV particles from Po-213 from the ranges.

The depth profiles of some of these longest trajectories are shown in Fig. 5B. The average range of these profiles was 25.7 ± 0.8 μm FWHM, while the simulated range was 28 μm. The Bragg peaks were not observed in the measured depth profiles. The lateral profiles, used to assess the spatial resolution of the trajectory images, are shown in Fig. 5C. The spatial resolution estimated from the trajectory width was 1.0 ± 0.1 μm FWHM. The uncertainty was estimated based on the standard deviations of the length or width of the measured profiles.

**Fig. 6:**
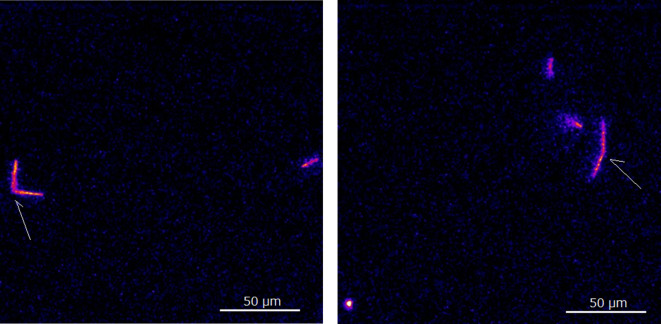
Alpha particle trajectory images contained alpha particles from Fr-221 (6.4 MeV) and At-217 (7.1 MeV) emitted almost simultaneously with “V” shape (indicated in arrow) measured with 100 ms and 40 x lens.

Figure 6 shows two particle trajectory images capturing alpha particles from Fr-221 (6.4 MeV) and At-217 (7.1 MeV) emitted from the same nucleus (but the different radionuclides), which were emitted nearly simultaneously, within a half-life of 32 ms. Each image contains “V”-shaped trajectories, representing alpha particles from Fr-221 and At-217 emitted from the same point. Approximately 12% (~ 20 out of 172 total images) of the measured images exhibited this characteristic “V”-shaped pattern. A video showing these “V”-shaped trajectories is provided in Supplemental Material-2. The video showed with the speed of 2 frames per sec to be observed details.

**Fig. 7:**
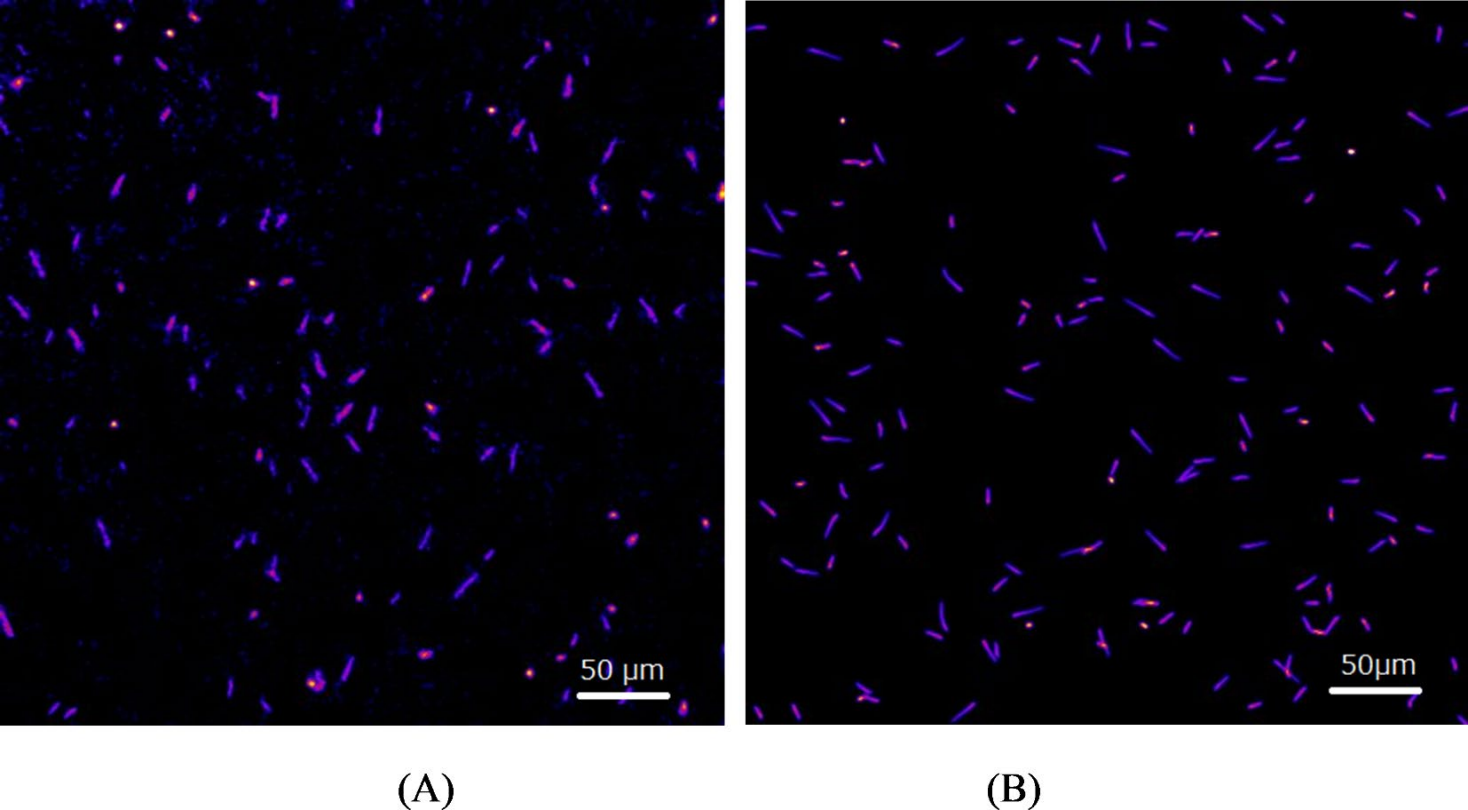
Measured image with 20 × lens of alpha-particles emitted from Ac-225 and its daughter radionuclides in GAGG plate (**A**) and simulated image (**B**).

Energy spectra were estimated from the measured images captured with a 20× lens of alpha particles emitted from Ac-225 and its daughter radionuclides in the GAGG plate. One of these images, used to evaluate the energy spectra, is shown in Fig. 7A. A simulated image with identical dimensions is shown in Fig. 7B, where similar alpha particle trajectories to those observed in the measured image can be seen.

**Fig. 8:**
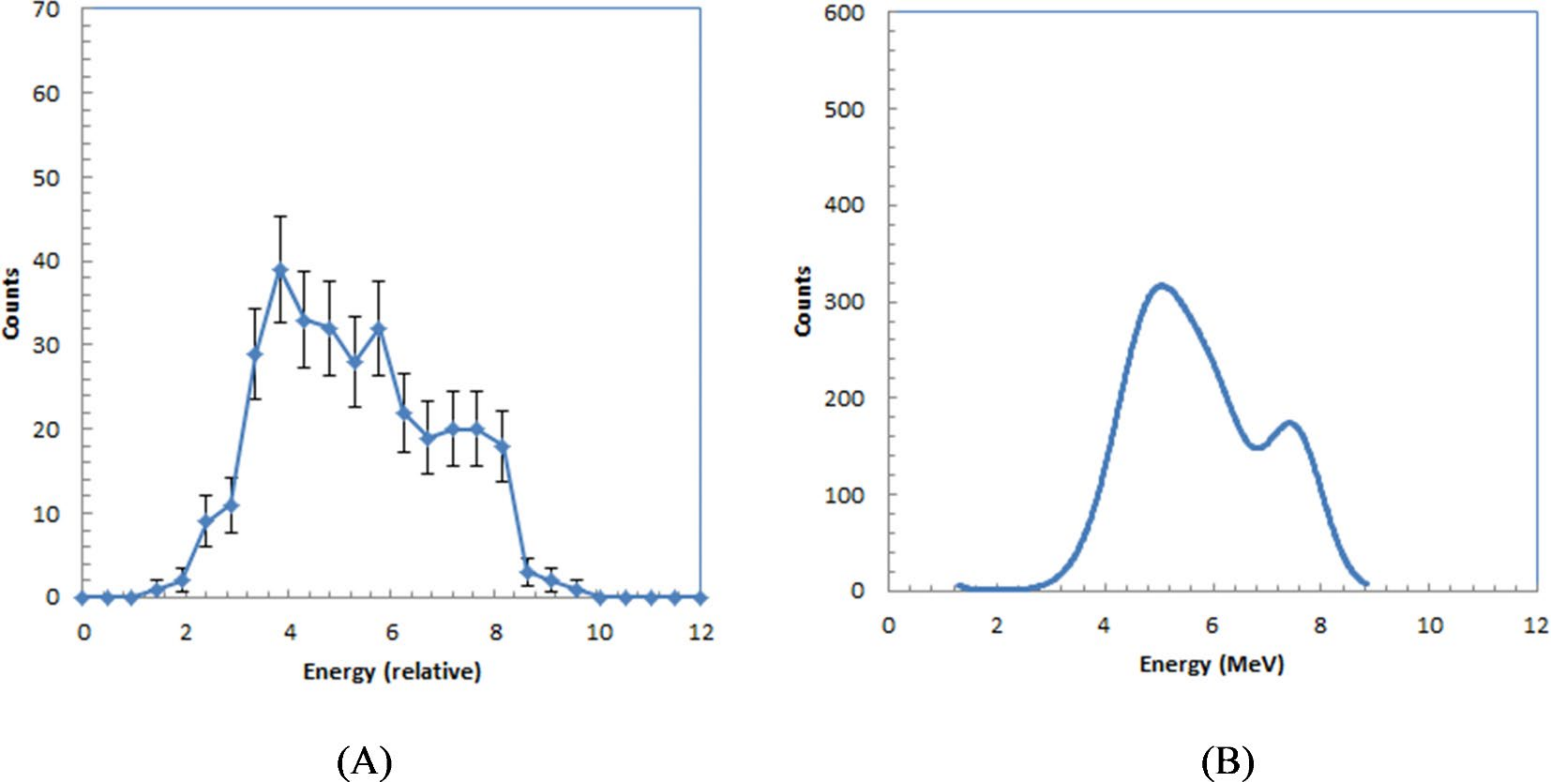
Energy spectra estimated from measured with 20 × lens of alpha-particles emitted from Ac-225 and its daughter radionuclides in GAGG plate (**A**) and smoothed energy spectra calculated by simulation (**B**).

Energy spectra estimated from the 500 ms acquisition time images, captured with a 20× lens, are presented in Fig. 8A. The spectra were obtained by defining regions of interest (ROIs) around approximately 300 trajectories to measure their intensities. The measured intensities were then used to plot a histogram of these values.

The smoothed energy spectrum calculated from the simulation is shown in Fig. 8B. The measured spectra exhibited a shape similar to the simulated spectra, suggesting that energy information can be extracted from the measured images by setting ROIs around the trajectories.

## Discussion

We successfully imaged the trajectories of alpha particles emitted by Ac-225 and its daughter radionuclides. Compared to the alpha-particle trajectory images obtained for Am-241^13^, some trajectories were notably longer. These extended trajectories are primarily due to the 8.4 MeV alpha particles from Po-213 traveling nearly parallel to the GAGG plate. The average measured range for the 8.4 MeV alpha particles was 25.7 μm, while the simulation-calculated range was 28 μm. The slightly shorter measured range is likely attributed to the incident angle of the alpha particles relative to the GAGG plate.

The spatial resolution of our alpha-particle imaging system (~ 1 μm) is significantly superior to that of traditional film-based systems utilizing CR-39^5^. The spatial resolution of CR-39 film is typically on the order of tens of micrometers, depending on factors such as etching time. The trajectories of alpha particles are generally elliptical or nearly circular, making accurate range or energy determination challenging with CR-39. In contrast, the high resolution achieved by our developed method enables determination of both ranges and energies. This makes it well-suited for imaging alpha-particle trajectories from Ac-225 and its daughter radionuclides within individual cells or tissue slices, especially in applications requiring ultra-high precision.

Another major advantage of our system is its capability for near real-time imaging. This short-interval imaging is particularly valuable for detecting the two alpha particles emitted by Fr-221 and At-217 within their 32 ms half-life, allowing us to capture particles from the same nucleus but different radionuclides. Furthermore, by incorporating ambient light, optical images of the subject can be captured. This capability will aid in identifying the cells or tissue slices that emitted alpha particles, and merged images of optical and alpha particle trajectories will be achieved. Additionally, it may enable the observation of dynamic morphological changes in specimens, such as cells, induced by alpha-particle irradiation.

The Bragg peaks were not observed in the measured depth profiles of alpha particles shown in Fig. 5B, likely due to the scintillator’s non-proportional response to alpha particles^21^. As the energy of the alpha particles decreased, the scintillator’s light output per MeV also diminished^21^, resulting in reduced light production in the Bragg peak regions and thus the absence of observable Bragg peaks. In the simulated trajectory images shown in Fig. 7B, the Bragg peaks were clearly visible. This is because the scintillator’s non-proportional response was not accounted for in the Monte Carlo simulation used^18^. Incorporating non-proportionality in the scintillation process within Monte Carlo simulations remains challenging because the non-proportional response to alpha particles is not yet fully understood and varies across different types of scintillators^21^. The trajectory images of 8.4 MeV alpha particles from Po-213 obtained in these experiments could provide valuable insights into the non-proportionality response of GAGG scintillators over a broad range of alpha particle energies.

By analyzing the intensities of the trajectory images, we derived the energy spectra of Ac-225 and its daughter radionuclides, as shown in Fig. 8A. The energy information from these trajectory images enables the identification of radionuclides emitting the alpha particles. However, the energy peaks corresponding to the four alpha particles from Ac-225 and its daughter radionuclides were not clearly separated. This overlap in the measured spectra could be due to light loss, potentially caused by the limited solid angle of the lens used in the imaging system. Employing a lens with higher light collection efficiency may improve the energy resolution of the alpha spectra.

Since the alpha particle trajectories we measured were projected as 2-dimensional images, depth information was not directly captured. However, the shapes and intensities of the trajectories contained some depth-related clues. For example, a high-intensity, short trajectory was likely due to particles entering nearly perpendicular to the scintillator plate. This orientation increases the observed intensity because the scintillation photons overlap when viewed from the camera side.　Additionally, such events often exhibited blurring in parts of the trajectories due to the off-focus effect at deeper sections^22^. By training a neural network with a combination of simulated 3-dimensional trajectory distributions and 2-directional projected images, it may be possible to reconstruct 3-dimensional trajectory distributions from the measured 2-directional projected images using deep learning^23^.

## Conclusions

We successfully captured the trajectories of alpha particles emitted by Ac-225 and its daughter radionuclides. These trajectory images, acquired with a spatial resolution of 1 μm, coupled with energy information and a temporal resolution of 100 ms, represent a significant advancement in the study of Ac-225 and its decay products. This high-resolution and real-time imaging system provides valuable insights, paving the way for a deeper understanding and more precise applications of Ac-225 in targeted alpha-particle therapy and related fields.

## Electronic supplementary material

Below is the link to the electronic supplementary material.

Supplementary Material 1

Supplementary Material 2

## Data Availability

Data is provided within the manuscript or supplementary information files.
